# Clinical and epidemiologic factors associated with breast cancer and its subtypes among Northeast Chinese women

**DOI:** 10.1002/cam4.2589

**Published:** 2019-10-23

**Authors:** Dong‐Man Ye, Qiang Li, Tao Yu, Hao‐Tian Wang, Ya‐Hong Luo, Wen‐Qing Li

**Affiliations:** ^1^ Department of Medical Imaging Liaoning Cancer Hospital & Institute Cancer Hospital of China Medical University Shenyang P. R. China; ^2^ Key Laboratory of Carcinogenesis and Translational Research (Ministry of Education/Beijing) Department of Cancer Epidemiology Peking University Cancer Hospital & Institute Beijing P. R. China; ^3^ Department of pathology Liaoning Cancer Hospital & Institute Cancer Hospital of China Medical University Shenyang P. R. China; ^4^ The First Clinical College Dalian Medical University Dalian P. R. China

**Keywords:** advanced stage, breast cancer, early stage, molecular subtypes

## Abstract

The incidence of breast cancer has increased dramatically in China. We evaluated the clinical and epidemiologic factors associated with breast cancer, and its stage in a case‐control study of Northeast Chinese women. We also examined whether these factors were differentially distributed among molecular subtypes of breast cancer in a case‐only analysis. We identified 1118 breast cancer patients and 2284 healthy women from Cancer Hospital of Medical University between January 2014 and December 2017. Logistic regression models were used to calculate the odds ratios (ORs) and corresponding 95% confidence intervals (CIs). We found that postmenopausal women had a decreased risk of breast cancer (multivariate‐adjusted OR = 0.33, 95% CI:0.25‐0.43), and tended to have breast cancer of human epidermal growth factor receptor 2 (HER2)‐overexpressing (multivariate‐adjusted OR = 2.99, 95% CI: 1.49‐5.97) and triple‐negative (multivariate‐adjusted OR = 2.16, 95% CI: 1.02‐4.56) subtypes, compared with the luminal B subtype. Women with history of abortion had an increased risk of breast cancer (multivariate‐adjusted OR = 4.70, 95% CI: 3.60‐6.14). Women with high breast density and high Breast Imaging Reporting and Data System (BIRADS) scores of lesions tended to have breast cancer of advanced stage, but were not differentially distributed among its molecular subtypes. In conclusion, postmenopausal women had decreased risk of breast cancer, and tended to have nonluminal subtype, while women with history of abortion had increased risk of breast cancer. Women with high breast density and BIRADS scores of lesions tended to have advanced stage breast cancer. We provide evidence on the epidemiologic factors for breast cancer and its subtypes, which may help with breast cancer risk stratification.

## Graphical abstract

Our study evaluated the clinical epidemiologic factors associated with breast cancer, and its stage and molecular subtypes in a case‐control study of Northeast Chinese women. We provide evidence on the epidemiologic factors associated with breast cancer stage and molecular subtypes, which may help with breast cancer risk stratification.

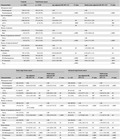

## 1 INTRODUCTION

Breast cancer is a major burden on women' health, with about 2.1 million newly diagnosed female cases in 2018 worldwide.^1^ A number of studies have evaluated the epidemiologic factors of breast cancer in Western countries, which reported aging, early menarche, late menopause, family history, lower parity, and alcohol consumption as major risk factors.^2^, ^3^, ^4^, ^5^, ^6^

The epidemiology of breast cancer and the related risk factors differ across countries and ethnicities.^7^ Previous studies have shown that breast cancer in Asian women has some unique features in epidemiologic risk factors. For example, breast cancer is associated with earlier ages at onset among Asian than Western populations.^8^ In China, the incidence of breast cancer has increased more than twice since 1990s, and the number of cases is estimated to reach 2.5 million overall by 2021.^9^ Although studies have examined the risk factors of breast cancer in Asia or specifically in China,^9^ efforts are still warranted to delineate the full spectrum of risk factors for the dramatically increased incidence. In addition, the epidemiology of breast cancer displays marked heterogeneities across regions of China.^10^ Among them, the Northeast China is a geographically and environmentally unique area, which has higher incidence (35.2 per 100 000) and mortality (6.8 per 100 000) of breast cancer than some other regions of China.^11^ Clarification of risk factors of breast cancer in Northeast Chinese women would be crucial for the development of prevention and management strategies. However, few studies have been conducted on the risk factors of breast cancer in Northeast China.

Breast cancer is a highly heterogeneous disease with various molecular subtypes, including the luminal A, luminal B, human epidermal growth factor receptor 2 (HER2)‐overexpressing, and triple‐negative subtypes.^12^ The epidemiological characteristics associated with molecular subtypes of breast cancer may be different, given their discrepant protein expression or genetic characteristics as well as the differential clinical outcomes.^13^ However, evidence on the epidemiologic risk factors for the heterogeneous subtypes of breast cancer is still limited for Chinese women, particularly in Northeast China.

In a case‐control study, we evaluated how the clinical epidemiologic factors changed the risk of breast cancer overall, and its clinical stage. To further investigate whether these risk factors influenced differently on stages and molecular subtypes of breast cancer among Northeast Chinese women, a case‐only analysis was conducted. In a secondary analysis, as high breast density is an established risk factor for the progression of breast cancer^14^ and Asian women have higher breast density compared to Western population, we also evaluated the clinical epidemiologic factors for breast density.

## 2 MATERIALS AND METHODS

### 2.1 Study participants

Our study included 1118 women with primary breast cancer who were identified from the Cancer Hospital of China Medical University from January 2014 to December 2017, including 497 early stage (stage I or II) and 621 advanced stage (stage III or IV) breast cancers. The diagnoses of breast cancer were pathologically confirmed based on breast cancer surgery or core needle biopsy. A total of 2284 healthy women undergoing a breast cancer screening program in the Cancer Hospital of China Medical University, supported by the National Cancer Center of China, during the same period were included as controls. Patients with other cancers or major cardiovascular diseases were excluded. Our study was approved by the Medical Ethical Committee of Liaoning Cancer Hospital and Institute. All the patients were informed and consented to use their general characteristics and clinical data.

## 3 DATA COLLECTION OF MAJOR CHARACTERISTICS

General characteristics of study participants, including age, menopausal status and age at menopause, personal history of abortion overall, and the history of spontaneous or induced abortion, were collected through face‐to‐face interviews by trained nurses at the Cancer Hospital. The information on general characteristics of cases was collected at diagnosis, while information on controls was collected at enrollment.

Information on breast density was obtained from the mammographic report. Breast density was classified into four categories,^15^ including “Almost entirely fat” with <25% glandular tissue (category I), “Scattered fibroglandular densities” with about 25%‐50% glandular tissue (category II), “Heterogeneously dense” breast with about 51%‐75% glandular tissue (category III), and “Extremely dense” breast with >75% glandular tissue (category IV). The category assessment of breast lesions was defined according to the radiographical features as shown in the Breast Imaging Reporting and Data System (BIRADS),^15^, ^16^ based on mammography. Because of the dense breast tissue of Chinese women, in addition to mammography, ultrasound was also used as a complementary tool to obtain the BIRADS scores of lesions.^16^ We missed the information on mammographic BIRADS scores for 379 participants and missed the information on ultrasound BIRADS scores for 213 participants. To differentiate benign and malignant lesions according to imaging features, mammographic and ultrasound BIRADS scores ranging from 0 to 6 were assigned^15^ to those with incomplete imaging which need additional imaging evaluation (a score of 0), negative findings (score 1), typically benign findings with essentially 0% likelihood of malignancy (score 2), probably benign findings with ≤2% risk of malignancy (score 3), suspicious abnormality with >2% but <95% risk of malignancy (score 4), highly suggestive of malignancy with ≥95% risk of malignancy (score 5), and known biopsy‐proven malignancy (score 6), respectively. The score of 0 was not available in our dataset, so the scores (1‐6) were re‐classified into three categories (1‐3, 4, or 5‐6) for sample size consideration.

Major clinical and pathological characteristics of the participants were also obtained. The location of breast cancers, including upper outer quadrant (UOQ), lower outer quadrant (LOQ), upper inner quadrant (UIQ), lower inner quadrant (LIQ), or central zone, was determined based on radiographical reports. Immunohistochemistry (IHC) assays of breast tissues were conducted to determine the status of estrogen receptor (ER), progesterone receptor (PR), HER2, and Ki67. The positive ER (ER+) or PR (PR+) status was defined as the positive nuclear staining present among 1% or more of tumor cells.^17^ HER2 staining was classified according to the percentage of positively stained tumor cell nuclei and the intensity of nuclear staining, with “−” for no staining, “1+” for weak intensity, “2+” for intermediate intensity, and “3+” for strong intensity.^18^ The categories of “−” or “1+” were categorized as HER2‐negative expression (HER2−) and “3+” as positive expression (HER2+). For tumors with “2+,” fluorescence in situ hybridization (FISH) was further conducted to identify the status of HER2. The percentage of Ki67‐positive cancer nuclei was categorized with 14% immunostained cells as the cutoff.^19^ The molecular subtypes of breast cancer, including luminal A (ER + and/or PR+, HER2–, and Ki67 < 14%), luminal B (ER+ and/or PR+, HER2−, and Ki67 ≥ 14% or ER+ and/or PR+, HER2+), HER2‐overexpressing (ER−, PR−, and HER2+), and triple‐negative (ER−, PR−, and HER2−) subtypes, were defined.^12^, ^19^

### 3.1 Statistical analysis

We firstly analyzed whether the major questionnaire‐based characteristics and breast density changed the risk of breast cancer overall, breast cancer of early stage, and advanced stage, respectively, as compared with the control group. Logistic regression models were used to calculate the odds ratios (ORs) and corresponding 95% confidence intervals (CIs). In the multivariable model, analyses were conducted including age (continuous variable), menopausal status, and age at menopause (premenopausal, age at menopause <50 years, age at menopause 50‐55, or age at menopause ≥55 years), history of abortion (never or had), and breast density (I, II, III, or IV). For breast density, category II, instead of category I, was used as the reference due to sample size consideration. We did not adjust for history of abortion for the separate analyses of spontaneous abortion (never or had) or induced abortion (never or had).

In addition to case‐control comparisons, case‐only analyses were conducted to examine whether these questionnaire‐based characteristics, breast density, and tumor location influenced differentially the risk for molecular subtypes of breast cancer, with luminal B breast cancer as the reference group. We defined molecular subtypes of breast cancer based on recognized methods,^12^, ^19^ including 73 cases of luminal A subtype, 398 cases of luminal B subtype, 89 cases of HER2‐overexpressing subtype, and 72 cases of triple‐negative subtype. Moreover, we also evaluated whether the same variables as described above changed the odds of advanced stage breast cancer, with early stage breast cancer as the reference. In addition, we were interested in understanding how ER, PR, HER2, and Ki‐67 expressions, the major determinants of breast cancer molecular subtypes, influenced the risk of advanced stage vs early stage breast cancer in Northeast China. Multivariate‐adjusted logistic regression models of case‐only analyses were used including the major questionnaire‐based characteristics, breast density, and tumor location.

We evaluated if mammographic and ultrasound BIRADS scores would be useful in predicting the stages and molecular subtypes of breast cancer. The analyses were conducted also with early stage or luminal B breast cancer as the reference group, respectively, adjusting for the major questionnaire‐based characteristics, breast density, and tumor location.

In a secondary analysis, the clinical epidemiologic factors for breast density were also examined based on all cases and controls with data on breast density (n = 3019). For this analysis, breast density category I was used as the reference.

All the statistical analyses were performed using SPSS 23.0. *P*‐value less than .05 was considered as statistically significant. All the results of statistical analyses were two‐sided.

## 4 RESULTS

### 4.1 Risk factors for breast cancer: a case‐control study

Baseline characteristics of the included breast cancer cases and the controls are shown in Table 1. Cases (mean age (SD): 57.0 (5.1) years) were significantly younger than controls (mean age (SD): 51.1 (8.2) years). Compared with premenopausal women, postmenopausal women had decreased risk of breast cancer of both early (multivariate‐adjusted OR = 0.30, 95% CI: 0.21‐0.42) and advanced stage (multivariate‐adjusted OR = 0.42, 95% CI: 0.31‐0.59). Among postmenopausal women, those with age at menopause more than 55 years had particularly higher risk of breast cancer (multivariate‐adjusted OR = 1.97, 95% CI: 1.19‐3.25) than other age groups at menopause. Women with a history of abortion had increased risk of breast cancer for both early stage (multivariate‐adjusted OR = 6.32, 95% CI: 4.57‐8.75) and advanced stage (multivariate‐adjusted OR = 3.23, 95% CI: 2.30‐4.53). Specifically, women with a history of spontaneous abortion, but not induced abortion, were at increased risk of breast cancer (multivariate‐adjusted OR = 2.29, 95% CI: 1.71‐3.07), especially for the early stage breast cancer (multivariate‐adjusted OR = 3.92, 95% CI: 2.78‐5.53). We also observed women with high breast density had increased risk of breast cancer, particularly for advanced stage (*P*_trend_ < .0001). Compared with breast density of category II (scattered), the multivariate‐adjusted OR (95% CI) for advanced stage breast cancer was 0.59 (0.33‐1.05) for women with category I (fatty), 2.11 (1.58‐2.80) for women with category III (heterogeneous), and 3.03 (1.69‐5.42) for women with category IV (extreme) (*P* for trend < .0001, Table 1). No such statistically significant trend was found for early stage breast cancer.

**Table 1:** The ORs (95% CI) for risk of breast cancer according to major characteristics

Characteristics	Controls	Total breast cancer				
	n = 2284	n = 1118	Age‐adjusted OR (95% CI)	*P*‐value	Multivariate‐adjusted OR (95% CI)^a^	*P*‐value
Menopausal status^b^						
Premenopausal	254 (11.1%)	552 (51.1%)	1.00		1.00	
Postmenopausal	2029 (88.9%)	529 (48.9%)	0.26 (0.21‐0.33)	<.0001	0.33 (0.25‐0.43)	<.0001
Age at menopause^c^						
<50 y	744 (36.7%)	190 (37.7%)	1.00		1.00	
50‐55 y	1200 (59.2%)	273 (54.2%)	1.03 (0.83‐1.27)	.80	0.97 (0.74‐1.26)	.81
≥55 y	83 (4.1%)	41 (8.1%)	2.93 (1.92‐4.47)	.0002	1.97 (1.19‐3.25)	.008
*P*_trend_				.40		.73
History of abortion						
Never	2130 (93.3%)	774 (72.8%)	1.00		1.00	
Had	152 (6.7%)	289 (27.2%)	5.23 (4.14‐6.60)	<.0001	4.70 (3.60‐6.14)	<.0001
History of spontaneous abortion^d^						
Never	2130 (93.3%)	859 (82.4%)	1.00		1.00	
Had	152 (6.7%)	183 (17.6%)	2.91 (2.27‐3.74)	<.0001	2.29 (1.71‐3.07)	<.0001
History of induced abortion^d^						
Never	2131 (93.3%)	946 (90.8%)	1.00		1.00	
Had	151 (6.6%)	96 (9.2%)	1.40 (1.05‐1.88)	.02	1.35 (0.96‐1.89)	.08
Breast density						
I (Fatty)	397 (17.4%)	38 (5.2%)	0.59 (0.40‐0.87)	.01	0.76 (0.51‐1.13)	.18
II (Scattered)	1069 (46.8%)	193 (26.3%)	1.00		1.00	
III (Heterogeneous)	772 (33.8%)	464 (63.1%)	2.32 (1.89‐2.85)	<.0001	1.85 (1.47‐2.31)	<.0001
IV (Extreme)	46 (2.0%)	40 (5.4%)	2.45 (1.50‐3.99)	.0003	2.04 (1.21‐3.46)	.0077
*P*_trend_				<.0001		<.0001

Characteristics	Early stage breast cancer					Advanced stage breast cancer				
	n = 497	Age‐adjusted OR (95% CI)	*P*‐value	Multivariate‐ adjusted OR (95% CI)^a^	*P*‐value	n = 621	Age‐adjusted OR (95% CI)	*P*‐value	Multivariate‐ adjusted OR (95% CI)^a^	*P*‐value
Menopausal status^b^										
Premenopausal	262 (53.6%)	1.00		1.00		290 (49.0%)	1.00		1.00	
Postmenopausal	227 (46.4%)	0.28 (0.22‐0.38)	<.0001	0.30 (0.21‐0.42)	<.0001	302 (51.0%)	0.29 (0.22‐0.38)	<.0001	0.42 (0.31‐0.59)	<.0001
Age at menopause^c^										
<50 y	91 (40.6%)	1.00		1.00		99 (35.4%)	1.00		1.00	
50‐55 y	114 (50.9%)	0.86 (0.64‐1.16)	.32	0.90 (0.62‐1.30)	.56	159 (56.8%)	1.01 (0.77‐1.33)	.94	1.04 (0.74‐1.46)	.82
≥55 y	19 (8.5%)	2.55 (1.45‐4.50)	.001	2.23 (1.10‐4.52)	.03	22 (7.8%)	2.08 (1.23‐3.52)	.007	1.86 (0.99‐3.51)	.05
*P*_trend_			.08		.23			.73		.44
History of abortion										
Never	309 (63.3%)	1.00		1.00		465 (80.9%)	1.00		1.00	
Had	179 (36.7%)	7.57 (5.73‐10.00)	<.0001	6.32 (4.57‐8.75)	<.0001	110 (19.1%)	3.33 (2.49‐4.45)	<.0001	3.23 (2.30‐4.53)	<.0001
History of spontaneous abortion^d^										
Never	351 (72.2%)	1.00		1.00		508 (91.4%)	1.00		1.00	
Had	135 (27.8%)	5.13 (3.84‐6.85)	<.0001	3.92 (2.78‐5.53)	<.0001	48 (8.6%)	1.27 (0.88‐1.84)	.20	1.11 (0.72‐1.70)	.65
History of induced abortion^d^										
Never	442 (90.9%)	1.00		1.00		504 (90.6%)	1.00		1.00	
Had	44 (9.1%)	1.24 (0.84‐1.83)	.06	1.16 (0.74‐1.82)	.53	52 (9.4%)	1.48 (1.04‐2.12)	.03	1.39 (0.92‐2.10)	.12
Breast density										
I (Fatty)	22 (6.6%)	0.74 (0.45‐1.22)	.24	1.01 (0.60‐1.69)	.98	16 (4.0%)	0.46 (0.26‐0.81)	.01	0.59 (0.33‐1.05)	.08
II (Scattered)	94 (28.2%)	1.00		1.00		99 (24.6%)	1.00		1.00	
III (Heterogeneous)	207 (62.2%)	2.08 (1.57‐2.76)	<.0001	1.49 (1.10‐2.03)	.01	257 (63.9%)	2.50 (1.91‐3.26)	<.0001	2.11 (1.58‐2.80)	<.0001
IV (Extreme)	10 (3.0%)	1.33 (0.63‐2.80)	.45	0.96 (0.42‐2.17)	.91	30 (7.5%)	3.49 (2.01‐6.06)	<.0001	3.03 (1.69‐5.42)	<.0001
*P*_trend_			<.0001		.06			<.0001		<.0001

Abbreviations: CI, confidence interval; OR, odds ratio.

a Unless otherwise noted, multivariable‐adjusted analyses were conducted including age (continuous variable), menopausal status and age at menopause (premenopausal, age at menopause <50 y, age at menopause 50‐55, or age at menopause ≥55 y), history of abortion (never or had), and breast density (I, II, III, or IV) in the model.

b All covariates as listed in footnote (a) but menopausal status and age at menopause were adjusted for.

c Analyses were conducted only in postmenopausal women.

d All covariates as listed in footnote (a) but history of abortion was adjusted for.

### 4.2 Comparison of major characteristics by stages and molecular subtypes of breast cancer: a case‐only analysis

We further examined whether these characteristics as well as tumor location changed the risk of advanced stage vs early stage breast cancer; consistent with the results shown in Table 1, postmenopausal women and women with higher breast density tended to have increased odds of advanced stage breast cancer, while women with a history of abortion, particularly spontaneous abortion, tended to have decreased odds of advanced stage breast cancer. In multivariate‐adjusted analyses, the LOQ (OR = 0.67, 95% CI: 0.47‐0.98), UIQ (OR = 0.60, 95% CI: 0.41‐0.87), and central zone (OR = 0.70, 95% CI: 0.50‐0.98) were less likely to have advanced stage breast cancer. We further examined ER, PR, Ki‐67, and HER2 status for the risk of advanced stage vs early stage breast cancer, and found that negative expression of PR (OR = 1.43, 95% CI: 1.02‐2.02), positive expression of HER2 (OR = 1.83, 95% CI: 1.29‐2.59), and the expression of Ki67 higher than 14% (OR = 1.58, 95% CI: 1.00‐2.50) increased the odds of breast cancer at advanced stage in multivariate‐adjusted analyses. (Table 2).

**Table 2:** The ORs (95% CI) for breast cancer at advanced stage vs early stage according to major clinical and pathological characteristics

Characteristics	Early stage	Advanced stage	Age‐adjusted OR (95% CI)	*P*‐value	Multivariate‐adjusted OR (95% CI)^a^	*P*‐value
Menopausal status^b^						
Premenopausal status	262 (53.6%)	290 (49.0%)	1.00		1.00	
Postmenopausal status	227 (46.4%)	302 (51.0%)	1.45 (1.02‐2.05)	.02	1.53 (1.06‐2.19)	.02
Age at menopause^c^						
Age at menopause <50 y	91 (40.6%)	99 (35.4%)	1.00		1.00	
Age at menopause 50‐55 y	114 (50.9%)	159 (56.8%)	1.12 (0.76‐1.65)	.57	1.12 (0.74‐1.68)	.59
Age at menopause ≥55 y	19 (8.5%)	22 (7.8%)	0.84 (0.42‐1.70)	.64	0.95 (0.45‐1.98)	.88
*P*_trend_				.92		.78
History of abortion						
Never	309 (63.3%)	465 (80.9%)	1.00		1.00	
Had	179 (36.7%)	110 (19.1%)	0.39 (0.30‐0.52)	<.0001	0.37 (0.28‐0.50)	<.0001
History of spontaneous abortion^d^						
Never	351 (72.2%)	508 (91.4%)	1.00		1.00	
Had	135 (27.8%)	48 (8.6%)	0.23 (0.16‐0.33)	<.0001	0.22 (0.15‐0.31)	<.0001
History of induced abortion^d^						
Never	442 (90.9%)	504 (90.6%)	1.00		1.00	
Had	44 (9.1%)	52 (9.4%)	1.07 (0.70‐1.64)	.76	1.06 (0.68‐1.65)	.79
Breast density						
I (Fatty)	22 (6.6%)	16 (4.0%)	0.66 (0.32‐1.37)	.27	0.68 (0.32‐1.46)	.33
II (Scattered)	94 (28.2%)	99 (24.6%)	1.00		1.00	
III (Heterogeneous)	207 (62.2%)	257 (63.9%)	1.16 (0.81‐1.65)	.42	1.25 (0.86‐1.82)	.23
IV (Extreme)	10 (3.0%)	30 (7.5%)	2.59 (1.18‐5.68)	.02	2.57 (1.14‐5.79)	.02
*P*_trend_				.02		.01
Tumor location						
Upper outer quadrant	152 (31.1%)	245 (40.6%)	1.00		1.00	
Lower outer quadrant	94 (19.3%)	101 (16.7%)	0.68 (0.48‐0.97)	.04	0.67 (0.47‐0.98)	.04
Upper inner quadrant	93 (19.1%)	88 (14.6%)	0.59 (0.41‐0.84)	.004	0.60 (0.41‐0.87)	.01
Lower inner quadrant	21 (4.3%)	26 (4.3%)	0.84 (0.45‐1.58)	.59	1.00 (0.51‐1.94)	1.00
Central zone	128 (26.2%)	143 (23.8%)	0.70 (0.51‐0.96)	.03	0.70 (0.50‐0.98)	.04
ER						
+	320 (74.8%)	183 (67.5%)	1.00		1.00	
‐	108 (25.2%)	88 (32.5%)	1.47 (1.05‐2.06)	.03	1.37 (0.96‐1.97)	.09
PR						
+	285 (67.1%)	155 (57.4%)	1.00		1.00	
‐	140 (32.9%)	115 (42.6%)	1.56 (1.13‐2.14)	.01	1.43 (1.02‐2.02)	.04
HER2						
‐	246 (62.8%)	121 (48.4%)	1.00		1.00	
+	146 (37.2%)	129 (51.6%)	1.81 (1.31‐2.52)	.0004	1.83 (1.29‐2.59)	.0007
Ki‐67						
<14%	86 (20.2%)	37 (14.0%)	1.00		1.00	
≥14%	339 (79.8%)	227 (86.0%)	1.62 (1.05‐2.49)	.03	1.58 (1.00‐2.50)	.05

Abbreviations: CI, confidence interval; OR, odds ratio; SD, standard deviation.

a Unless otherwise noted, multivariable‐adjusted analyses were conducted including age (continuous variable), menopausal status and age at menopause (premenopausal, age at menopause <50 y, age at menopause 50‐55, or age at menopause ≥55 y), history of abortion (never or had), breast density (I, II, III, or IV), and tumor location (upper outer quadrant, lower outer quadrant, upper inner quadrant, lower inner quadrant, or central zone) in the model.

b All covariates as listed in footnote (a) but menopausal status and age at menopause were adjusted for.

c Analyses were conducted only in postmenopausal women.

d All covariates as listed in footnote (a) but history of abortion was adjusted for.

For the analyses of molecular subtypes (Table 3), postmenopausal women tended to have breast cancer of HER2‐overexpressing (multivariate‐adjusted OR = 2.99, 95% CI: 1.49‐5.97) and triple‐negative (multivariate‐adjusted OR = 2.16, 95% CI: 1.02‐4.56) subtypes. We did not find a trend in the risk of these subtypes of breast cancer with the increased age at menopause (multivariate‐adjusted *P*_trend_ = .90 for HER2‐overexpressing and *P*_trend_ = .50 for triple‐negative breast cancer). Relative to luminal B subtype, breast cancers in the central zone were less likely to be HER2‐overexpressing subtype (multivariate‐adjusted OR = 0.48, 95% CI: 0.24‐0.94), and breast cancers in the UIQ were more likely to be luminal A subtypes (multivariate‐adjusted OR = 2.44, 95% CI: 1.20‐4.99). History of abortion and breast density were not differentially associated with molecular subtypes of breast cancer.

**Table 3:** The ORs (95% CI) for molecular subtypes of breast cancer by major characteristics

Characteristics	Luminal B	Luminal A				
	n=398	n=73	Age‐adjusted OR (95% CI)	*P*‐value	Multivariate‐ adjusted OR (95% CI)^a^	*P*‐value
Menopausal status^b^						
Premenopausal	211 (54.6%)	36 (51.4%)	1.00		1.00	
Postmenopausal	175 (45.4%)	34 (48.6%)	0.66 (0.31‐1.38)	.28	0.64 (0.29‐1.39)	.26
Age at menopause^c^						
<50 y	62 (37.6%)	15 (44.1%)	1.00		1.00	
50‐55 y	94 (57.0%)	16 (47.1%)	0.67 (0.30‐1.50)	.33	0.63 (0.27‐1.48)	.29
≥55 y	9 (5.4%)	3 (8.8%)	1.30 (0.31‐5.52)	.72	1.63 (0.33‐8.12)	.55
*P*_trend_				.71		.80
History of abortion						
Never	254 (66.0%)	49 (70.0%)	1.00		1.00	
Had	131 (34.0%)	21 (30.0%)	0.82 (0.47‐1.43)	.48	0.80 (0.45‐1.42)	.44
History of spontaneous abortion^d^						
Never	292 (76.8%)	53 (76.8%)	1.00		1.00	
Had	88 (23.2%)	16 (23.2%)	0.98 (0.53‐1.81)	.95	0.99 (0.53‐1.87)	.98
History of induced abortion^d^						
Never	341 (89.7%)	65 (94.2%)	1.00		1.00	
Had	39 (10.3%)	4 (5.8%)	0.53 (0.18‐1.55)	.25	0.49 (0.16‐1.47)	.20
Breast density						
I (Fatty)	14 (5.0%)	2 (3.8%)	0.68 (0.14‐3.31)	.63	0.55 (0.11‐2.86)	.48
II (Scattered)	82 (29.2%)	17 (32.7%)	1.00		1.00	
III (Heterogeneous)	168 (59.8%)	30 (57.7%)	1.10 (0.53‐2.24)	.80	1.07 (0.51‐2.25)	.86
IV (Extreme)	17 (6.0%)	3 (5.8%)	1.18 (0.29‐4.79)	.81	1.31 (0.31‐5.54)	.71
*P*trend				.86		.82
Tumor location						
Upper outer quadrant	123 (31.7%)	20 (27.8%)	1.00		1.00	
Lower outer quadrant	76 (19.6%)	8 (11.0%)	0.72 (0.30‐1.76)	.48	0.71 (0.29‐1.76)	.46
Upper inner quadrant	66 (17.0%)	22 (30.6%)	2.24 (1.12‐4.50)	.02	2.44 (1.20‐4.99)	.01
Lower inner quadrant	18(4.6%)	4 (5.6%)	1.51 (0.45‐5.01)	.50	1.79 (0.52‐6.19)	.36
Central	105 (27.1%)	18 (25.0%)	1.14 (0.56‐2.30)	.73	1.21 (0.59‐2.49)	.60

Characteristics	HER2‐overexpressing					Triple‐negative				
	n=89	Age‐adjusted OR (95% CI)	*P*‐value	Multivariate‐ adjusted OR (95% CI)^a^	*P*‐value	n=72	Age‐adjusted OR (95% CI)	*P*‐value	Multivariate‐ adjusted OR (95% CI)^a^	*P*‐value
Menopausal status^b^										
Premenopausal	33 (37.1%)	1.00		1.00		34 (47.2%)	1.00		1.00	
Postmenopausal	56 (62.9%)	2.90 (1.48‐5.70)	.002	2.99 (1.49‐5.97)	.002	38 (52.8%)	2.03 (0.97‐4.25)	.06	2.16 (1.02‐4.56)	.05
Age at menopause^c^										
<50 y	24 (45.3%)	1.00		1.00		17 (48.6%)	1.00		1.00	
50‐55 y	26 (49.1%)	0.81 (0.42‐1.58)	.53	0.77 (0.37‐1.61)	.49	15 (42.9%)	0.65 (0.29‐1.43)	.28	0.61 (0.26‐1.43)	.61
≥55 y	3 (5.6%)	1.01 (0.25‐4.14)	.99	1.34 (0.31‐5.86)	.70	3 (8.5%)	1.38 (0.33‐5.86)	.66	0.90 (0.19‐4.22)	.90
*P*_trend_			.80		.90			.81		.50
History of abortion										
Never	62 (72.1%)	1.00		1.00		43 (61.4%)	1.00		1.00	
Had	24 (27.9%)	0.76 (0.45‐1.28)	.30	0.78 (0.45‐1.34)	.37	27 (38.6%)	1.24 (0.73‐2.11)	.42	1.35 (0.77‐2.36)	.29
History of spontaneous abortion^d^										
Never	65 (77.4%)	1.00		1.00		48 (70.6%)	1.00		1.00	
Had	19 (22.6%)	0.97 (0.55‐1.70)	.91	0.97 (0.54‐1.75)	.89	20 (29.4%)	1.38 (0.78‐2.46)	.27	1.38 (0.76‐2.51)	.19
History of induced abortion^d^										
Never	79 (94.0%)	1.00		1.00		62 (91.2%)	1.00		1.00	
Had	5 (6.0%)	0.58 (0.22‐1.51)	.26	0.61 (0.23‐1.65)	.34	6 (8.8%)	0.90 (0.36‐2.22)	.81	0.93 (0.37‐2.35)	.88
Breast density										
I (Fatty)	2 (3.3%)	0.59 (0.12‐2.86)	.51	0.63 (0.13‐3.12)	.57	1 (1.8%)	0.37 (0.05‐3.07)	.36	0.33 (0.04‐2.77)	.31
II (Scattered)	21 (34.4%)	1.00		1.00		16 (28.6%)	1.00		1.00	.47
III (Heterogeneous)	35 (57.4%)	1.00 (0.52‐1.92)	1.00	1.02 (0.52‐2.02)	.95	33 (58.9%)	0.90 (0.44‐1.81)	.76	0.77 (0.37‐1.59)	.34
IV (Extreme)	3 (4.9%)	0.87 (0.22‐3.44)	.85	0.87 (0.21‐3.56)	.84	6 (10.7%)	1.65 (0.53‐5.16)	.39	1.77 (0.54‐5.76)	.17
*P*_trend_			.89		.80			.26		.27
Tumor location										
Upper outer quadrant	36 (42.4%)	1.00		1.00		21 (29.2%)	1.00		1.00	
Lower outer quadrant	18 (21.2%)	0.77 (0.40‐1.47)	.42	0.76 (0.39‐1.48)	.42	29 (40.3%)	0.61 (0.29‐1.30)	.20	0.57 (0.26‐1.23)	.15
Upper inner quadrant	13 (15.3%)	0.66 (0.33‐1.35)	.27	0.74 (0.36‐1.54)	.43	12 (16.6%)	0.53 (0.23‐1.22)	.13	0.60 (0.25‐1.44)	.26
Lower inner quadrant	3 (3.5%)	0.39 (0.08‐1.75)	.22	0.41 (0.09‐1.92)	.26	8 (11.1%)	0.48 (0.11‐2.21)	.35	0.44 (0.09‐2.07)	.30
Central	15 (17.6%)	0.47 (0.24‐0.92)	.02	0.48 (0.24‐0.94)	.03	2 (2.8%)	0.74 (0.39‐1.42)	.36	0.78 (0.40‐1.52)	.46

Abbreviations: CI, confidence interval; OR, odds ratio.

a Unless otherwise noted, multivariable‐adjusted analyses were conducted including age (continuous variable), menopausal status and age at menopause (premenopausal, age at menopause <50 y, age at menopause 50‐55, or age at menopause ≥55 y), history of abortion (never or had), breast density (I, II, III, or IV), and tumor location (upper outer quadrant, lower outer quadrant, upper inner quadrant, lower inner quadrant, or central zone) in the model.

b All covariates as listed in footnote (a) but menopausal status and age at menopause were adjusted for.

c Analyses were conducted only in postmenopausal women.

d All covariates as listed in footnote (a) but history of abortion was adjusted for.

The BIRADS scores of mammography and ultrasound were also evaluated for the risk of breast cancer stages and molecular subtypes (Table 4). Women with high BIRADS scores of mammography and ultrasound tended to have breast cancer at advanced stage vs early stage (both multivariate‐adjusted *P*_trend_ < .0001), but were not differentially distributed among molecular subtypes.

**Table 4:** The ORs (95% CI) for different stage and molecular subtypes of breast cancer according to mammographic and ultrasound BIRADS scores of lesions

Characteristics	Mammographic BIRADS scores of lesions				Ultrasound BIRADS scores of lesions			
	1‐3	4	5‐6	*P* for trend	1‐3	4	5‐6	*P* for trend
Stage of breast cancer								
Early stage (n)	24	192	192		13	139	334	
Advanced stage (n)	28	274	29		46	188	185	
Age‐adjusted OR (95% CI)	1.38 (0.76‐2.50)	1.00	9.69 (6.23‐15.07)		0.39 (0.20‐0.75)	1.00	2.59 (1.94‐3.46)	
*P*‐value	.29		<.0001	<.0001	.005		<.0001	<.0001
Multivariate‐ adjusted OR (95% CI)^a^	1.57 (0.84‐2.93)	1.00	10.60 (6.64‐16.93)		0.39 (0.19‐0.77)	1.00	2.94 (2.15‐4.01)	
*P*‐value	.16		<.0001	<.0001	.007		<.0001	<.0001
Molecular subtypes of breast cancer								
Luminal B (n)	19	180	83		23	130	201	
Luminal A (n)	3	37	12		4	29	28	
Age‐adjusted OR (95% CI)	0.50 (0.11‐2.27)	1.00	0.68 (0.34‐1.39)		0.80 (0.26‐2.51)	1.00	0.65 (0.37‐1.16)	
*P*‐value	.37		.29	.82	.70		.14	.47
Multivariate‐adjusted OR (95% CI)^a^	0.56 (0.12‐2.64)	1.00	0.79 (0.38‐1.64)		0.82 (0.25‐2.69)	1.00	0.68 (0.38‐1.24)	
*P*‐value	.46		.53	.97	.75		.21	.57
HER2‐overexpressing subtype (n)	4	40	16		3	30	43	
Age‐adjusted OR (95% CI)	0.97 (0.31‐3.03)	1.00	0.88 (0.46‐1.68)		0.57 (0.16‐2.04)	1.00	0.95 (0.56‐1.60)	
*P*‐value	.96		.71	.95	.39		.85	.59
Multivariate‐adjusted OR (95% CI)^a^	0.99 (0.30‐3.24)	1.00	0.84 (0.43‐1.65)		0.65 (0.17‐2.42)	1.00	0.91 (0.53‐1.56)	
*P*‐value	.99		.61	.86	.52		.73	.95
Triple‐negative subtype (n)	1	35	20		2	28	22	
Age‐adjusted OR (95% CI)	0.30 (0.04‐2.30)	1.00	1.35 (0.73‐2.52)		0.41 (0.09‐1.82)	1.00	0.78 (0.44‐1.37)	
*P*‐value	.25		.34	.06	.24		.39	.77
Multivariate‐adjusted OR (95% CI)^a^	0.31 (0.04‐2.46)	1.00	1.22 (0.64‐2.33)		0.52 (0.11‐2.48)	1.00	0.82 (0.46‐1.47)	
*P*‐value	.27		.55	.08	.41		.51	.78

Abbreviations: CI, confidence interval; BIRADS, breast imaging reporting and data system; OR, odds ratio.

a Multivariable‐adjusted analyses were conducted adjusting for age (continuous variable), menopausal status and age at menopause (premenopausal, age at menopause <50 y, age at menopause 50‐55, or age at menopause ≥55 y), history of abortion (never or had), breast density (I, II, III, or IV), and tumor location (upper outer quadrant, lower outer quadrant, upper inner quadrant, lower inner quadrant, or central zone) in the model.

### 4.3 Major characteristics for breast density: a secondary analysis

Consistent with the direction of how these factors changing breast cancer risk, we found lower breast density for postmenopausal women and higher breast density for women with a history of abortion, as shown in Table S1. A history of both spontaneous and induced abortion increased the odds of higher breast density (Table S1). In addition, higher age at menopause increased the odds of higher breast density (Table S1).

## 5 DISCUSSION

Based on the Northeast Chinese women, we comprehensively evaluated the epidemiologic factors for breast cancer and its clinical stage and molecular subtypes. We found that postmenopausal women had a decreased risk of breast cancer, and tended to have breast cancer of advanced stage as well as HER2‐overexpressing and triple‐negative subtypes, while a history of abortion had an increased risk of breast cancer. Women with a high breast density and BIRADS scores of lesions tended to have advanced stage breast cancer compared with early stage breast cancer, but were not differentially distributed among the molecular subtypes of breast cancer.

Previous studies have examined the association between menopausal status and risk of breast cancer. A meta‐analysis including 118 964 women with breast cancer and 306 091 women without breast cancer showed that premenopausal women had 40% higher risk of breast cancer than postmenopausal women at the age of 45 to 54 years.^2^ A multicenter cross‐sectional study in China also reported that two‐thirds of breast cancer cases were diagnosed before menopause.^20^ In our study, we found that postmenopausal women had decreased odds of breast cancer. In addition, we observed lower breast density among postmenopausal women, while breast density is a major risk factor for breast cancer.^14^, ^21^ Among postmenopausal women, we found that age at menopause of more than 55 years, compared with other age groups at menopause, had high breast density and increased odds of breast cancer, consistent with other studies based on Chinese women.^22^, ^23^ Menarche and menopause represent the start and end of ovarian and endocrine activity related to reproduction, respectively.^2^ Early menarche and late menopause may reflect long‐time exposure to estrogen and progesterone, which have been recognized for increased breast density and breast cancer risk.^24^, ^25^ Previous studies have shown that estrogen and progesterone could induce the growth, division, and proliferation of breast cells,^26^ and also increase the probability of a random genetic error and the susceptibility to carcinogens.^24^ The molecular mechanisms underlying the findings also remain elucidated. For example, plasma insulin‐like growth factor‐I and insulin‐like growth factor binding protein‐3 levels have been shown to underlie the associations between higher breast density and increased breast cancer risk only among premenopausal women, but not among postmenopausal women.^27^

Interestingly, although postmenopausal women overall had a reduced risk of breast cancer, in our study, postmenopausal women tended to have late stage breast cancer relative to early stage breast cancer, and were more likely to have HER2‐overexpressing (ER‐, PR−, and HER2+) and triple‐negative subtypes (ER−, PR−, HER2−), the two breast cancer subtypes with worse prognosis.^28^ Previous studies among Chinese women have examined the associations between menopausal status and various combinations of molecular markers of breast cancer. A recent study consisting of 8067 Chinese women focused on the same molecular subtypes of breast cancer with us and demonstrated that postmenopausal women tended to have HER2‐overexpressing and triple‐negative subtypes.^29^ Also consistent with our study, another study on Northeast Chinese women found that postmenopausal status decreased the odds of luminal A and luminal B subtypes compared to controls.^30^ Therefore, previous studies based on Chinese population in different regions showed generally consistent results with our findings. This is biologically plausible given the recognized mechanism of estrogen and progesterone exposure underlying ER+ and PR+ breast cancer, while the development of ER− and PR− breast cancer may be independent of female hormones.^31^

In our study, the association between a history of abortion, particularly spontaneous abortion, and increased risk of breast cancer may be biologically plausible. The theory that a full‐term pregnancy is at decreased risk of breast cancer has been generally accepted.^32^ Exposure to the pregnancy hormones is required for breast cells to complete the differentiation, which is crucial for the lowered susceptibility of breast cells to carcinogenesis in a women's later life,^33^, ^34^ whereas the process of differentiation can be interrupted by abortions. However, previous epidemiological studies provided inconsistent evidence about the association between abortion and breast cancer. A collaborative reanalysis summarizing 53 epidemiological studies, including studies on Chinese and Western women, did not support spontaneous or induced abortion associated with the risk of breast cancer.^35^ Another meta‐analysis among Chinese women reported that a history and the increased frequency of induced abortion were associated with increased risk of breast cancer.^36^ Among the six studies based on Northeastern Chinese women included in this meta‐analysis,^36^ the positive association between induced abortion and breast cancer was reported in four studies, but other two studies did not find so. The exact reason for the distinct results between our study and prior Chinese studies is still unclear, which requires further studies to clarify.

Consistent with the direction of changing breast cancer risk, women with history of abortion had increased breast density. However, it is worth noting that although women with both spontaneous and induced abortion had higher breast density, a history of spontaneous abortion only, but not induced abortion, increased the risk of breast cancer overall. Previous studies on abortion and breast density have been sparse.^37^, ^38^ We also examined a history of abortion for clinical stage and molecular subtypes of breast cancer. Interestingly, women with a history of abortion were more likely to be diagnosed with early stage breast cancer, but were not associated with its molecular subtypes. Few studies have evaluated abortion for clinical stage of breast cancer, but studies on abortion and molecular subtypes reported controversial findings. A study based on Iranian women found that a history of abortion was associated with luminal B breast cancer compared to other subtypes.^39^ Another study among Northeastern Chinese women reported that spontaneous abortion was inversely associated with luminal A and luminal B subtypes, while induced abortion was associated with increased risk of luminal A tumors.^30^ Further researches are warranted to clarify the effect of abortion on the stage and molecular subtypes of breast cancer and the potential underlying mechanisms.

Our study based on Northeast Chinese women supported that women with high breast density had elevated risk of breast cancer, particularly advanced stage breast cancer. However, breast density was not differentially distributed among molecular subtypes of breast cancer in our study, which appears inconsistent with a prior US study showing increased mammographic density for HER2‐positive breast cancer.^40^ Whether the heterogeneity in results may be attributed to the disparities between Chinese and Western population is unknown, and further research would be needed.

In addition to menopausal status, history of abortion, and breast density, we further examined whether the status of ER, PR, HER2, and Ki‐67 as well as tumor location would change the odds of late stage vs early stage breast cancer. Compared with early stage breast cancer, women with ER‐, PR‐, HER2+, and high expression of Ki‐67 (≥14%) had increased odds of advanced stage breast cancer, consistent with the previous reports indicating worse clinical outcomes for these immunohistochemical categories.^41^, ^42^, ^43^ The association between primary tumor location and patient's prognosis has been evaluated previously for progression of breast cancer.^44^, ^45^, ^46^, ^47^, ^48^ In our study, we found that breast tumors located in the UOQ and LIQ tended to be advanced stage breast cancer. Similarly, two prior studies found that breast cancers in the LIQ were associated with a shorter overall survival among Chinese women,^44^, ^47^ which can be explained by a higher rate of internal mammary lymph node metastasis for LIQ tumors.^49^ We further found that relative to luminal B subtype, luminal A breast cancer tended to occur in the UIQ, but HER2‐overexpressing breast cancer was less likely to occur in the central zone. The inner (LIQ, UIQ) and central zones have been related to worse overall breast cancer survival or disease‐free survival in several previous studies.^45^, ^46^, ^48^ However, we did not find a tendency of these breast zones to have triple‐negative or HER2‐overexpressing breast cancers, the two subtypes with the worse prognosis. In contrast, a Korean study reported more frequently diagnosed HER2‐overexpressing subtype in the LIQ and triple‐negative subtype in the UOQ zone, which appeared generally consistent with our findings on the advanced stage breast cancer in LIQ and UOQ.^46^ Collectively, research on breast cancer tumor location and its clinical stage and molecular subtypes has not reached all consistent findings. The different findings within our own study and between studies in various settings may reflect the complexity of breast cancer. The clinical features of breast cancer may be attributed to a series of characteristics with tumor location as one factor. Further studies are still needed.

We placed particular emphasis on the implications of breast imaging examination and evaluated whether the BIRADS scores would be different for the clinical stage and molecular subtypes of breast cancer. As expected, women with high BIRADS scores tended to have advanced stage breast cancer compared with early stage breast cancer in our study, demonstrating potentially greater likelihood of malignant behavior of breast cancer. The results appeared similar for ultrasound and mammographic scores. However, we did not find differences in BIRADS scores among breast cancer molecular subtypes, suggesting that BIRADS scores may not serve as predictors for molecular subtypes of breast cancer.

Our study was strengthened by its extensive investigations on major epidemiologic factors, breast density, tumor location, and BIRADS scores for breast cancer and its clinical stage and molecular subtypes based on the Northeast Chinese women. In addition, we explored major characteristics for breast density. Our study also had limitations. First, our study was a hospital‐based unmatched case‐control study with its intrinsic limitations. Selection bias may be a concern as the breast cancer patients were selected from the Cancer Hospital of China Medical University, and controls were selected from a breast cancer screening program in this hospital. The cases were significantly younger than controls. However, the mean age of both case and control categories was older than 50 years. We have adjusted for age (as a continuous variable instead of categorical variable) in all the statistical analyses. We believe our results of case‐control analyses were less likely to be distorted even for the known age‐related menopausal status and history of abortion, as the continuous age‐adjusted analyses were able to minimize the effects of unbalanced age distribution. However, the potential extrapolation of our findings to other settings might need caution. Second, information on postmenopausal status and age at menopause as well as history of abortion were self‐reported, which may have led to information bias, although the misclassification would tend to be nondifferential among the different clinical stages and molecular subtypes of breast cancer. Third, our study did not collect information on several important hormonal and reproductive factors as well as other host and lifestyle factors of breast cancer, such as reproductive history, breastfeeding, oral contraceptive use, parity, number of abortion, family history of breast cancer, and body mass index. Further efforts are warranted to examine how these factors may change the risk of breast cancer and its subtypes in Northeast Chinese women and to examine whether any factor may contribute to explaining the inconsistency of our findings with prior epidemiologic studies. In addition, studies are also required to examine breast cancer subtypes defined by additional clinical factors, such as axillary nodal status, mitotic index, and nuclear pleomorphism. Fourth, we were not able to examine separately the urban and rural areas, or examine different regions and ethnicity groups altogether in our study, which requires further large scale multicentered studies.

In conclusion, we found that postmenopausal women had decreased risk of breast cancer, and tended to have breast cancer of HER2‐overexpressing and triple‐negative subtypes, while women with a history of abortion had increased risk of breast cancer. Women with a high breast density and mammographic and ultrasound BIRADS scores tended to have advanced stage breast cancer. Our study provides evidence on the epidemiologic factors for breast cancer stage and molecular subtypes, and may inform practitioners on the risk stratification of breast cancer in clinics.

## DISCLOSURE STATEMENT

The authors declare no potential conflict of interest.
