# Supplementary material for: Clinical and epidemiologic factors associated with breast cancer and its subtypes among Northeast Chinese women
Source: Cancer Med. 2019 Oct 23;8(17):7431–45. doi: 10.1002/cam4.2589 (PMC6885867; doi:10.1002/cam4.2589)
Supplement: Supplementary file 1 [file CAM4-8-7431-s001.docx]

IRB approval document
