# Supplementary material for: Clinical and epidemiologic factors associated with breast cancer and its subtypes among Northeast Chinese women
Source: Cancer Med. 2019 Oct 23;8(17):7431–45. doi: 10.1002/cam4.2589 (PMC6885867; doi:10.1002/cam4.2589)
Supplement: Supplementary file 2 [file CAM4-8-7431-s002.docx]

Supplementary Table S1. The ORs (95% CI) for categories of breast density according to major characteristics.

| Characteristics | I | II | | | | | III | | | | | IV | | | | |
| --- | --- | --- | --- | --- | --- | --- | --- | --- | --- | --- | --- | --- | --- | --- | --- | --- |
|  | n=435 | n=1262 | Age-  adjusted  OR  (95% CI) | P-value | Multivariate- adjusted  OR  (95% CI) ^†^ | P-value | n=1236 | Age-  adjusted  OR  (95% CI) | P-value | Multivariate- adjusted  OR  (95% CI) ^†^ | P-value | n=86 | Age-  adjusted  OR  (95% CI) | P-  value | Multivariate-adjusted  OR  (95% CI) ^†^ | P-value |
| **Menopausal status**^‡^ | |  |  |  |  |  |  |  |  |  |  |  |  |  |  |  |
| Premenopausal | 16  (3.7%) | 129  (10.3%) | 1.00 |  | 1.00 |  | 416  (34.0%) | 1.00 |  | 1.00 |  | 41  (48.8%) | 1.00 |  | 1.00 |  |
| Postmenopausal | 418  (96.3%) | 1128  (89.7%) | 0.37  (0.21-0.65) | 0.0006 | 0.39  (0.22-0.69) | 0.001 | 806  (66.0%) | 0.14  (0.08-0.24) | <0.0001 | 0.14  (0.08-0.25) | <0.0001 | 43  (51.2%) | 0.20  (0.08-0.45) | 0.0001 | 0.20  (0.09-0.48) | 0.0003 |
| **Age at menopause**^§^ | |  |  |  |  |  |  |  |  |  |  |  |  |  |  |  |
| <50 years | 195  (46.8%) | 407  (36.3%) | 1.00 |  | 1.00 |  | 254  (31.9%) | 1.00 |  | 1.00 |  | 10  (23.8%) | 1.00 |  | 1.00 |  |
| 50-55 years | 213  (51.1%) | 661  (59.0%) | 1.54  (1.22-1.94) | 0.0003 | 1.54  (1.22-1.95) | 0.0003 | 493  (62.0%) | 1.96  (1.52-2.53) | <0.0001 | 2.01  (1.55-2.60) | <0.0001 | 30  (71.4%) | 3.05  (1.42-6.52) | 0.004 | 3.25  (1.47-7.16) | 0.004 |
| ≥55 years | 9  (2.1%) | 53  (4.7%) | 3.08  (1.48-6.42) | 0.003 | 2.82  (1.35-5.90) | 0.006 | 48  (6.1%) | 6.04  (2.85-12.82) | <0.0001 | 6.48  (3.04-13.80) | <0.0001 | 2  (4.8%) | 8.77  (1.48-51.89) | 0.02 | 8.61  (1.38-53.68) | 0.02 |
| *P*_trend_ |  |  |  | <0.0001 |  | <0.0001 |  |  | <0.0001 |  | <0.0001 |  |  | 0.0011 |  | 0.0010 |
| **History of abortion** | |  |  |  |  |  |  |  |  |  |  |  |  |  |  |  |
| Never | 417  (96.1%) | 1145  (91.4%) | 1.00 |  | 1.00 |  | 994  (82.0%) | 1.00 |  | 1.00 |  | 74  (88.1%) | 1.00 |  | 1.00 |  |
| Had | 17  (3.9%) | 108  (8.6%) | 2.34  (1.38-3.95) | 0.002 | 2.07  (1.22-3.53) | 0.007 | 218  (18.0%) | 4.51  (2.69-7.58) | <0.0001 | 4.44  (2.62-7.53) | <0.0001 | 10  (11.9%) | 6.57  (2.13-20.23) | 0.001 | 5.06  (1.53-16.72) | 0.008 |
| **History of spontaneous abortion**^¶^ | | |  |  |  |  |  |  |  |  |  |  |  |  |  |  |
| Never | 422  (97.5%) | 1163  (93.0%) | 1.00 |  | 1.00 |  | 1041  (86.3%) | 1.00 |  | 1.00 |  | 76  (91.6%) | 1.00 |  | 1.00 |  |
| Had | 11  (2.5%) | 87  (7.0%) | 2.89  (1.53-5.47) | 0.001 | 2.61  (1.37-4.97) | 0.004 | 165  (13.7%) | 5.11  (2.72-9.60) | <0.0001 | 5.41  (2.85-10.26) | <0.0001 | 7  (8.4%) | 6.74  (1.83-24.75) | 0.004 | 6.36  (1.55-26.17) | 0.01 |

(Continued) Supplementary Table S1. The ORs (95% CI) for categories of breast density according to major characteristics.

| Characteristics | I | II | | | | III | | | | | | IV | | | | |
| --- | --- | --- | --- | --- | --- | --- | --- | --- | --- | --- | --- | --- | --- | --- | --- | --- |
|  | n=435 | n=1262 | Age-  adjusted  OR  (95% CI) | P-  value | Multivariate- adjusted  OR (95% CI) ^†^ | P-  value | n=1236 | Age-  adjusted  OR  (95% CI) | P-value | Multivariate- adjusted  OR (95% CI) ^†^ | P-value | n=86 | Age-  adjusted  OR  (95% CI) | P-value | Multivariate-adjusted  OR  (95% CI) ^†^ | P-value |
| **History of induced abortion**^¶^ | | |  |  |  |  |  |  |  |  |  |  |  |  |  |  |
| Never | 421  (97.2%) | 1177  (94.0%) | 1.00 |  | 1.00 |  | 1077  (89.3%) | 1.00 |  | 1.00 |  | 76  (91.6%) | 1.00 |  | 1.00 |  |
| Had | 12  (2.8%) | 75  (6.0%) | 2.30  (1.23-4.27) | 0.009 | 2.29  (1.23-4.26) | 0.01 | 129  (11.7%) | 4.19  (2.26-7.77) | <0.0001 | 4.15  (2.24-7.69) | <0.0001 | 7  (8.4%) | 9.26  (2.50-34.27) | 0.0009 | 9.81  (2.62-36.76) | 0.003 |

Breast density categories: I (Fatty), II (Scattered), III (Heterogeneous), IV (Extreme).

OR, odds ratio; CI, confidence interval; SD, standard deviation.

^†^ Unless otherwise noted, multivariable-adjusted analyses were conducted including age (continuous variable), menopausal status and age at menopause (premenopausal, age at menopause <50 years, age at menopause 50-55, or age at menopause ≥55 years), and history of abortion (never or had) in the model.

^‡^ All covariates as listed in footnote (a) but menopausal status and age at menopause were adjusted for.

^§^ Analyses were conducted only in postmenopausal women.

^¶^ All covariates as listed in footnote (a) but history of abortion were adjusted for.
